# Selection and perceived impact of walk-up songs in college baseball

**DOI:** 10.3389/fpsyg.2025.1543835

**Published:** 2025-03-21

**Authors:** Sarah Stokowski, Chris Corr, Michael Godfrey, Matthew Eric, Chase Hughes, Rob Hughes, Matthew Marchal, Taylor Roby

**Affiliations:** Department of Educational & Organizational Leadership Development, Clemson University, Clemson, SC, United States

**Keywords:** audial selection, musicality, cognitive enhancement, athletic performance, athletics, NCAA, fan engagement

## Abstract

**Introduction:**

Music and song have the ability to positively affect athletic performance. Music has been demonstrated to increase physical capabilities and improve cognitive function among both recreational and competitive athletes. This study sought to explore the role of music on athletic performance by examining walk-up songs in competitive athletics.

**Methods:**

A sample of 10 participants currently competing in National Collegiate Athletic Association Division I major conference baseball agreed to participate in semi-structured qualitative interviews. Participants were asked to detail the process leading to their selection of a walk-up song and describe the perceived impact of walk-up songs on their athletic performance.

**Findings:**

Participants categorized three salient factors contributing to the selection of their walk-up songs: (1) personal preference, (2) crowd engagement, and (3) song popularity/relevance. In addition, participants indicated that they derived athletic performance benefits from their walk-up song selection based on the ability of a selected song to engage the crowd and narrow their focus prior to an at-bat.

**Discussion:**

Although participants indicated they selected their walk-up songs based primarily on personal preference, factors pertaining to the desires of participants to engage and appeal to the crowd undoubtedly impacted their walk-up song selection. In this sense, participants indicated the primary benefit derived from walk-up songs was in engaging the crowd in the game itself. Such finding is in contrast to extant scholarship identifying the role of music and song in individual athletic performance and perhaps indicative of the increased role fans occupy in athletic performance.

## 1 Introduction

Extant research illustrates the prescriptive role of music in improving sport and athletic performance (Akhshabi and Rahimi, 2021; Terry et al., 2020). In fact, music has been linked to increased physical exertion, oxygen consumption, and physical performance among athletes in both competitive and recreational sport settings. In addition to physical performance, music also possesses the ability to impact an individual’s cognitive state during athletic activity (Terry et al., 2020). As athletic performance is dictated by a combination of physical and mental congruity (Charest and Grandner, 2022), the role and effect of music in athletic performance is notable and significant.

Although many competitive sporting events incorporate music within a stadium or arena as a contributing component to an event’s general ambiance (Gaffney and Bale, 2004), few sports incorporate distinct customizable musical choices within athletic contests. Competitive baseball and softball, however, allow for competing athletes to select individual musical choices that accompany and preempt performance. Colloquially referenced as walk-up songs, these musical choices are selected by athletes themselves and befit individual players’ personalities. While walk-up songs are uncommon in many team sport settings, walk-up—or walk-out—songs are regularly integrated into combat sports (e.g., boxing, wrestling; Fowlkes, 2020). Given fans can establish an association between a specific song or genre of music with an individual athlete, walk-up songs are powerful mechanisms from a branding standpoint. However, the effect of music on an athlete’s performance is especially salient in the context of sport given the expressed emphasis on winning inherent to sport itself.

Although music is linked to increased athletic performance (Akhshabi and Rahimi, 2021; Terry et al., 2020) and walk-up songs are a mainstay in competitive sports such as baseball, softball, and combat sports, no research to date has explored the manner in which athletes select their walk-up songs. Accordingly, the present study sought to explore factors influencing athletes’ selection of walk-up songs as well as perceptions regarding the role and impact of walk-up songs on individual performance. Utilizing a sample of National Collegiate Athletic Association (NCAA) Division I baseball players, the researchers explored athletes’ selection and perceived impact of walk-up songs on performance. In accordance with the purpose of exploration, the following research questions were formulated to assist in the design of the study:

- Which factors impact how Division I baseball players select their walk-up songs?
- To what extent do Division I baseball players perceive walk-up songs impact their athletic performance?

## 2 Method

### 2.1 Arousal-mood-hypothesis

The arousal-mood-hypothesis can be utilized to explain the impacts and benefits of music within exercise and activity. Schaefer (2017) found that music can have a positive influence on arousal and mood and that music in active spaces initiates brain clusters that regulate emotions. Such emotional regulation from music resulting in positive arousal and mood can lead to an increase in physical output. In this sense, individuals have a positive reaction to specific and distinct music or musical genres. Such positive reactions among individuals are notable in the context of sport and athletics, specifically, given the physical and cognitive enhancement derived from exposure to music (He et al., 2017).

### 2.2 Sample of participants

To explore the selection and impact of walk-up music on athletic performance in competitive sport, the researchers utilized a sample of NCAA Division I athletes competing in *major* conference baseball. The major conference in NCAA Division I athletics represent the highest level of competition within collegiate athletics in the United States. Participants were recruiting through a combination of convenience and snowball sampling. Considering the insulated nature of collegiate athletics in the United States, securing participants is often a logistical challenge when conducting research on competing college athletes (Corr et al., 2024). Accordingly, the researchers relied on an initial convenience sample of four baseball players at two unique universities. Subsequent snowball sampling resulting in the recruitment of six additional participants. In total, 10 participants (*n* = 10) from four (*n* = 4) universities agreed to participate in this study. Subsequent efforts to secure participants failed to produce additional current NCAA Division I major conference baseball players willing to participate in the research. Purposively, each participant had participated in NCAA Division I major conference baseball for more than three seasons. Given only one of the participants had used the same walk-up song since their first competitive collegiate season, utilizing participants with three or more seasons experience in collegiate baseball was valuable to fully explore their unique selection of walk-up songs over time.

### 2.3 Procedure and analysis

Given that the researchers strived to understand the reasons players chose specific walk-up songs and how it impacted them, a phenomenological approach was deemed suitable to explore participants’ perspectives through descriptions of lived experiences as NCAA Division I baseball players. The researchers utilized a semi-structured interview protocol to explore both the salient factors contributing to the selection of individual participants’ walk-up songs and participants’ perceptions of the impact of their walk-up song on their athletic performance.

Upon agreeing to participate in the semi-structured interview, participants were provided an informed consent document. All 10 participants composing the findings of this study provided their informed consent and virtual video interviews were scheduled during the month of March 2024. Interviews were recorded and last an average of 34-min in length. Each interview was transcribed and inductively coded using in-vivo codes. Given the importance of member-checking to validity and reliability, participants were provided copies of the transcribed interviews prior to analysis. Notably, each participant indicated the transcripts were an accurate representation of their expressed opinions and no language was requested to be changed or contextualized differently.

The use of an inductive in-vivo coding schema was deemed appropriate considering the exploratory nature of the study. Each of the eight members of the research team participated in an open-coding process designed at identifying pertinent thematic areas pertaining to the salient factors influencing each participants walk-up song selection. Upon completion of initial coding, the researchers met as a group to compare notes and establish consensus among the coded thematic areas. The researchers, in aggregate, determine three primary factors most saliently impacted participants’ selection of walk-up songs: (1) personal preference, (2) crowd engagement, and (3) song popularity/relevance. The researchers conducted another round of open-coding to explore participants’ perceptions with regards to the impact of walk-up songs on athletic performance. Similarly, the researchers met after each performing a round of initial coding to compare notes and establish consensus. Based on the responses of participants, two perceived areas of impact were identified as the most salient among responses: (1) crowd engagement and (2) “locked in.”

### 2.4 Positionality and trustworthiness

To ensure transparency, a researcher positionality statement is necessary. The research team consisted of eight individuals: six males and two females. At the time of data collection, three individuals were participating NCAA Division I baseball players. In addition, one researcher is a former Division I softball player. While the presence of multiple current and former Division I athletes was an expressed benefit in the formulation of the study and the recruitment of participants, the researchers also readily acknowledge the need to avoid confirmation bias at any point while conducting this study. Accordingly, the necessary steps were taken to ensure reliability and trustworthiness of the data presented subsequently in the findings section. The researchers utilized a pilot study, bracketed interviews, and conducted coding and analysis as a collective group to ensure consistency and reliability of the findings. While the authors acknowledge the position of the researchers with regards to the sampled population, we would also note the value of such proximity to potential participants. Considering the insulated nature of collegiate athletics and the difficulty of securing participants from a population of college athletes (Corr et al., 2024), utilizing current institutional insiders was of strategic value to be able to conduct this study.

## 3 Findings

### 3.1 Walk-up song selection

Three factors emerged as the most salient components impacting participants’ selection of walk-up songs: (1) personal preference, (2) crowd engagement, and (3) song popularity/relevance. Among participants, personal preference was the most salient factor impacting the selection of a walk-up song. Participants’ personal preferences varied by musical genre or historical significance. Some participants indicated they selected walk-up songs based off the walk-up song of their favorite professional baseball player while others indicated that they used walk-up songs of former teammates. Other than personal preference, crowd engagement and song popularity/relevance were separate, yet related, factors most saliently impacted the selection of participants’ walk-up songs. Among participants, each indicated it was important that their walk-up song engaged the crowd in some meaningful way. Songs with musical beats conducive to clapping hands or stomping feet were mentioned as meaningful ways to engage fans while popular songs with lyrics widely known by traditional college-aged students were also a valuable mechanism in which to get fans engaged with the player and game. Some participants stated that athletic department personnel from marketing and/or communications departments worked with their respective baseball teams to select “fan friendly” or “engaging” walk-up songs. However, among each participant, the decision for walk-up song selection resided solely with the players on the team and tended to be made primarily out of personal preference.

### 3.2 Perceived impact of walk-up song

Among participants, two prominent themes emerged when discussing the perceived impact of walk-up song on athletic performance: (1) crowd engagement and (2) “locked in.” Most saliently, participants believed that their walk-up song contributed to engaging the crowd and creating a raucous and advantageous homefield environment. The term “atmosphere” was used frequently among participants to describe the impact of their walk-up song on engaging fans. Each of these terms fit within the components of *arousal* within the arousal-mood-hypothesis. Considering walk-up songs are only played during a team’s home games, the perceived importance of engaging fans with a motivational or catchy walk-up song is notable with regards to the perceived impact of walk-up songs on athletic performance. In this sense, an engaged crowd is the mechanism participants were drawing motivation from, rather than a specific walk-up song itself.

“Locked in” was identified as the second thematic area in which participants indicated walk-up songs contributed to their athletic performance. Participants indicated that their choice of walk-up song was an extension and component of their “locking in” prior to a plate appearance during a baseball game. Such indications among participants are consistent with extant literature indicating distinct songs or musical genres have the ability to increase cognitive attentiveness, which is positively correlated to improved athletic performance (Akhshabi and Rahimi, 2021; Terry et al., 2020).

## 4 Discussion

The present study sought to explore the factors contributing to competitive athletes’ selection of walk-up songs during athletic competition and explore the perceived impact of such music on athletic performance. From a sample of 10 participants currently competing in NCAA Division I major conference baseball, the findings of this study indicate that crowd engagement is among the most salient factors influencing walk-up song selection and of the greatest perceived influence on athletic performance. While participants indicated personal preference was the predominant factor impacting walk-up song selection, the secondary factors of crowd engagement and song popularity/relevance indicate that players may select walk-up songs for a variety factors based on what they perceive will engage fans in the game itself. Given participants stated they perceived crowd engagement to be the most impactful component of walk-up songs with regards to athletic performance, such factor influencing walk-up song selection is not surprising. As athletes seek to maximize their performance with sport, prioritizing crowd engagement and the perceived desires of fans is perhaps an intuitive psychological process for athletes when selecting a walk-up song. If the greatest perceived impact to athletic performance from a walk-up song is crowd engagement, athletes may naturally gravitate towards songs and musical genres they believe will engage fans. In the context of the arousal-mood-hypothesis (Schaefer, 2017), participants in this study indicated that crowd engagement was a form of arousal that directly impacted their mood. Such finding is interesting considering extant scholarship identifying distinct songs or musical genres impact on athletes’ performance (Akhshabi and Rahimi, 2021; Terry et al., 2020). While personal preferences is a salient factor impacting the selection of participants’ walk-up songs, the perceived impact of crowd engagement on performance is notable within an athletes’ cognitive process to select a walk-up song.

Of additional note were participants’ desires to connect with fans through the selection of their walk-up songs. In this sense, the indication that song popularity/relevance impacted participants’ selection of walk-up songs is perhaps intuitive as well. Seeking to connect with fans with current popular music is, perhaps, reflective of the desire to associate with and garner support of student peers. Posited in this manner, walk-up songs among participants were less of a reflection of their personal musical preference and more reflective of their desire to engage and connect with fans attending the game.

Contextually, it is interesting to note the selection of walk-up songs within the context of perceived improvement to athletic performance. Considering only half of a college baseball team’s games are played at a homefield, the role of walk-up songs is, further, less about the individual player and more about creating an environment or, in the specific words of numerous participants, “atmosphere.” As walk-up songs are only played during home games and players must also perform, inherently, without walk-up songs while playing games on the road and during postseason competition, the selection of a walk-up song for the purposes of engaging a crowd at home is of greater significance than the selection of a walk-up song for purely personal athletic performance enhancement. Such finding is notable in the context of walk-up song selection and the role of music within athletic performance if competitive athletes are selecting walk-up—or walk-out—songs for the expressed purposes engaging fans. While such fan engagement is *consumed* by athletes and channeled for athletic performance enhancement purposes, the logic for selection a walk-up song based on personal enjoyment or motivation is somewhat contradictory to extant scholarship examining the role and value of music to athletes and improved athletic performance.

## 5 Limitations and future research

With the context of walk-up songs in sports, collegiate baseball represents an extremely narrow population in which to research. In addition, the use of convenience sampling, while we believed justified, still serves as a limitation to this study. Future research examining a greater breadth of sports featuring walk-up songs would be valuable to further contextualize the findings of this study. While participants in this study indicated that walk-up songs were selected, predominantly, based in pursuit of environmental factors (e.g., crowd engagement, song popularity), future research in other sports where walk-up or walk-out songs are common would be valuable as an exploration to uncover the psychological factors contributing to athletes’ selection of walk-up songs.

## Data Availability

The original contributions presented in the study are included in the article/supplementary material, further inquiries can be directed to the corresponding author.
